# First Report of *Hartmannella* keratitis in a Cosmetic Soft Contact Lens Wearer in Iran

**Published:** 2013

**Authors:** Hoda ABEDKHOJASTEH, Maryam NIYYATI, Firoozeh RAHIMI, Mansour HEIDARI, Shohreh FARNIA, Mostafa REZAEIAN

**Affiliations:** 1 Department of Parasitology and Mycology, School of Public Health, Tehran University of Medical Sciences, Tehran, Iran; 2 Department of Medical Parasitology and Mycology, School of Medicine, Shahid Beheshti University of Medical Sciences, Tehran, Iran; 3 Farabi Eye Hospital, Tehran University of Medical Sciences, Tehran, Iran; 4 Department of Medical Genetics, Tehran University of Medical Sciences, Tehran, Iran; 5 Center for Research of Endemic Parasites of Iran (CREPI), Tehran University of Medical Sciences, Tehran, Iran

**Keywords:** *Hartmannella*, Amoebic keratitis, Contact lens, Iran

## Abstract

**Background:**

Poor hygiene will provide good condition for corneal infections by opportunistic free-living amoebae (FLA) in soft contact lens wearers. In the present study an amoebic keratitis due to *Hartmannella* has been recognized in a 22-year-old girl with a history of improper soft contact lens use. She had unilateral keratitis on her left eye. Her clinical signs were eye pain, redness, blurred vision and photophobia. The round cysts of free-living amoebae were identified in non-nutrient agar medium by light microscopy. These cysts were suspected to be *Hartmannella* using morphological criteria. A PCR assay has been confirmed that the round cysts were belonged to *H. vermiformis*.

## Introduction

Free-living amoebae (FLA) including *Acanthamoeba* spp., *Balamuthia mandrillaris, Naegleria fowleri, Hartmannella* spp. and *Vahlkampfia* are widely distributed in diverse environments including soil, water, water-air interface and dust (1). One important aspect of these FLA is their predatory function on bacteria which make them unique in balancing the biosphere (2). To date, keratitis due to FLA especially *Acanthamoeba* keratitis (AK) is increasing. AK is a painful eye infection caused by poor hygiene and improper lens handling in contact lens wearers and individuals with corneal trauma (3, 4). Swimming while wearing contact lens and homemade solutions are other risk factors related to *Acanthamoeba* keratitis (5).

There are different studies reporting other FLA, such as *Vahlkampfia* and *Hartmannella* as causal agents of amoebic keratitis (6, 7). *Hartmannella* genus is limax amoeba. Worm-like shape trophozoites of *H. vermiformis* are able to make round double-walled cysts (8). There is a previous report regarding the mixed infection of *Acanthamoeba* belonging to T3 genotype and Vahlkampfia in Iran (9). Although *Hartmannella* was classified as human parasite in 1997 (10), the pathogenicity of this genus in humans and animals is disputable (11–13). One important aspect of keratitis due to other FLA could be the poor response to anti-*Acant-hamoeba* treatment. Thus accurate diagnosis is important for successful treatment; otherwise it may cause corneal epithelium ulceration developing to perforation and even vision loss.

Here we report a case of amoebic keratitis due to *Hartmannella* genus using morphological and molecular based analysis. To the best of our knowledge this is the first report of *Hartmannella* keratitis in Iran.

## Case report

**Fig. 1:**
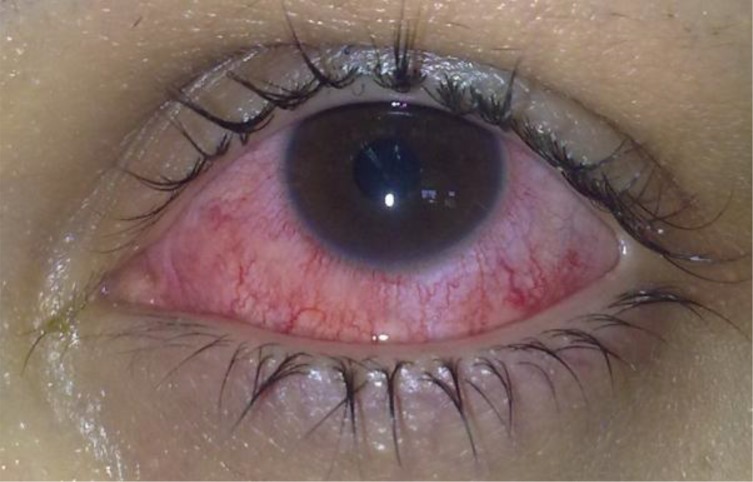
Clinical photograph of the patient at the admission time

A 22-year-old girl presented unilateral keratitis on her left eye with complaints of eye pain, redness (Fig. 1), blurred vision, light sensitivity, tearing and foreign body sensation. Patient was referred to the protozoology laboratory in Tehran University of Medical Sciences for further evaluation in April 2011. She was a cosmetic soft contact lenses wearer. On confocal scan of the cornea there were multifocal areas of mild nonspecific opacity in the subepithelial and anterior stromal regions. No typical cyst or trophozoite-like structures were present on confocal scans. The confocal scan features were not suggestive of an *Acanthamoeba* keratitis.

Before referral to our center, she was treated with ciprofloxacin eye drops (ciloxan^®^) and gentamicine. Since no notable improvement was seen after treatment by these antibiotics, it was deduced that keratitis should not be with bacterial etiology. The patient complained a stinging eye after administration the eye drops.

Contact lenses, storage case and cleaning solution were tested by culture on 1.5% non-nutrient agar covered with a layer of heat-inactivated *Escherichia coli* incubated at room temperature for 14 days and investigated daily by light microscopy.

**Fig. 2:**
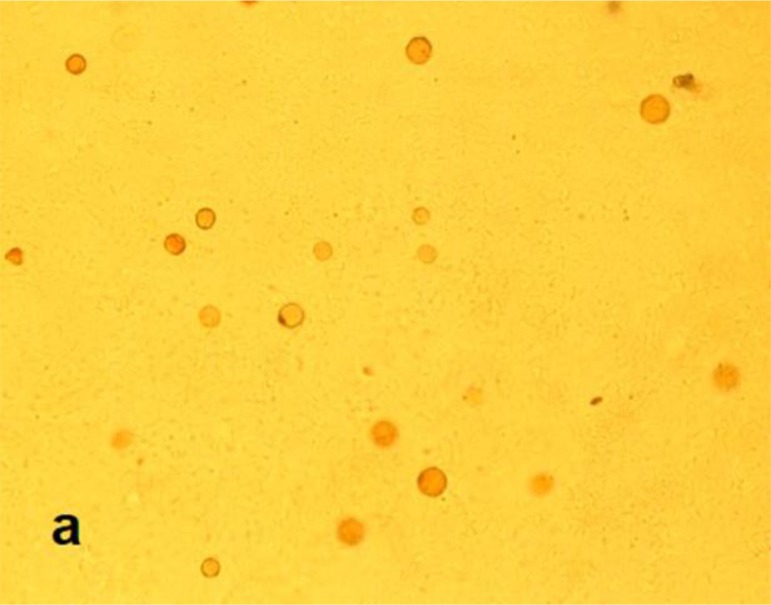
Light microscopy image of *Hartmannella vermiformis* cysts on non-nutrient agar (X400)

**Fig. 3:**
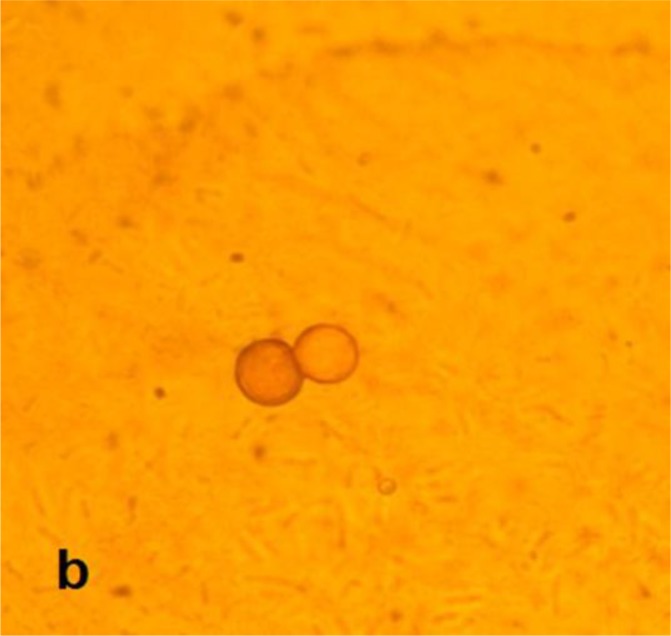
Light microscopy image of *Hartmannella vermiformis* cysts on non-nutrient agar (X1000)

Cultures of contact lenses and their cases were positive about one month after first inoculation on 1.5% non-nutrient agar. No bacterial and fungal agents were isolated. The rounded cysts (Figs. 2 and 3) and worm-shaped trophozoites were detected by light microscopy examinations. These rounded cysts were cloned and DNA extraction was done using phenol chloroform method (14). A PCR assay has been performed using NA primers with following sequences: NA1: 5′-GCT CCA ATA GCG TAT ATT AA-3′ and NA2: 5′-AGA AAG AGC TAT CAA TCT GT-3′ (15).

PCR was done in 30 µl Ampliqon (Taq DNA Polymerase Master Mix RED, Denmark). Twenty-five microliters of Taq Master Mix were used with 5 ng template DNA, 0.1 µM of each primer and water. The PCR cycling conditions were an initial denaturing step of 94°C for 1 min and 35 repetitions of denaturation at 94°C for 35 s, annealing at 50°C for 45 s, and extension at 72 °C for 1 min, with a final extension of 72 °C for 10 min. PCR products were electrophoresed on a 1.5% agarose gel and visualized under UV light. Sequencing and BLAST analysis has revealed that this sequence was belonged to *H. vermiformis*.

*Pathogenicity* determination of the amoebae was carried out by temperature tolerance test on non-nutrient agar covered with heat-inactivated *Escherichia coli* at 37, 40, and 45°C temperatures. After 72 hours the samples were examined by light microscopy. The cysts were differentiated to the trophozoites at 37 °C but not 40 °C and 45 °C.

The patient was treated with *polyhexamethylene biguanide* (PHMB) 0.02% for two weeks. After one year of following up the patient, she was satisfied with treatment and all her clinical signs were totally recovered.

## Discussion

The first *Acanthamoeba* eye infection has been reported in UK in 1974 (16). Since then AK has been reported with increasing rate by developing modern techniques in parallel with contact lens wearers. The frequency rate of *Acanthamoeba* keratitis cases are steadily promoting in Iran (17). During recent years not only *Acanthamoeba* eye infection but also mixed infections by other FLA have been increasingly reported. Although the pathogenicity of other FLA including *Vahlkampfia and Hartmannella* (11, 18) is not approved yet, they are supposed to implicate in human keratitis (19). Other studies reported *Vahlkampfia* and *Hartmannella* strains are able to perform pathogenicity on human keratocytes as performed by *Acanthamoeba* strains (20, 21). Niyyati et al. reported the first case of amoebic mixed infection due to *Acanthamoeba* genotype T3 and *Vahlkampfia* in a cosmetic soft contact lens wearer in Iran (9). As keratitis in this case showed poor response to anti-*Acanthamoeba* treatment, they considered other FLA as potential agents of keratitis. Lorenzo-Morales et al. presented a mixed infection by *Acanthamoeba* genotype T4 and *Hartmannella* species in a 21-year-old man with background of soft contact lens use (22). The early diagnosis of mixed infection leaded to favorable response to treatment. The present research showed that the patient is suffering from *Hartmannella* keratitis. No other FLA has been detected in the culture of the aforementioned patient. This is an important finding since it can conclude that at least some *Hartmannella* strains are able to cause keratitis. Inoue et al reported a mixed *Acanthamoeba* – *Hartmannella* keratitis in a 54-year-old woman with background of hard contact lens use which had responded poorly to anti-amoebic drugs. Poor response was referred to *Hartmannella* coexistence in corneal ulcer (18).

Thermotolerance test was performed at various temperatures in that trophozoites just could grow at 37 °C after excystation. Replication at this temperature noted that the isolate has the potential pathogenicity in human as a real host.

In this case we have reported a young woman with amoebic keratitis due to *H. vermiformis*. This is the first report regarding the occurrence of *Hartmannella* in a contact lens wearer in Iran. The present study reflects that other free-living amoebae could be an agent of keratitis and accurate diagnosis of free-living amoebae should be done by morphological and molecular methods.
